# The Development of Shared Liking of Representational but not Abstract Art in Primary School Children and Their Justifications for Liking

**DOI:** 10.3389/fnhum.2016.00021

**Published:** 2016-02-05

**Authors:** Paul Rodway, Julie Kirkham, Astrid Schepman, Jordana Lambert, Anastasia Locke

**Affiliations:** Department of Psychology, University of ChesterChester, UK

**Keywords:** empirical aesthetics, convergence, semantic association, neurocognitive development, meta-cognition, emotion

## Abstract

Understanding how aesthetic preferences are shared among individuals, and its developmental time course, is a fundamental question in aesthetics. It has been shown that semantic associations, in response to representational artworks, overlap more strongly among individuals than those generated by abstract artworks and that the emotional valence of the associations also overlaps more for representational artworks. This valence response may be a key driver in aesthetic appreciation. The current study tested predictions derived from the semantic association account in a developmental context. Twenty 4-, 6-, 8- and 10-year-old children (*n* = 80) were shown 20 artworks (10 representational, 10 abstract) and were asked to rate each artwork and to explain their decision. Cross-observer agreement in aesthetic preferences increased with age from 4–8 years for both abstract and representational art. However, after age 6 the level of shared appreciation for representational and abstract artworks diverged, with significantly higher levels of agreement for representational than abstract artworks at age 8 and 10. The most common justifications for representational artworks involved subject matter, while for abstract artworks formal artistic properties and color were the most commonly used justifications. Representational artwork also showed a significantly higher proportion of associations and emotional responses than abstract artworks. In line with predictions from developmental cognitive neuroscience, references to the artist as an agent increased between ages 4 and 6 and again between ages 6 and 8, following the development of Theory of Mind. The findings support the view that increased experience with representational content during the life span reduces inter-individual variation in aesthetic appreciation and increases shared preferences. In addition, brain and cognitive development appear to impact on art appreciation at milestone ages.

## Introduction

Evaluating an object’s beauty is a central property of human behavior with aesthetic preferences developing in early infancy (Krentz and Earl, 2013), and influencing behavior in a wide range of circumstances. Understanding how aesthetic judgements develop and what determines aesthetic appreciation is an important challenge for psychologists (Gardner et al., 1975; Lin and Thomas, 2002; Leder et al., 2004; Göksun et al., 2014; for a review, see Lindell and Mueller, 2011). A primary focus of this research is an examination, from a developmental perspective, of the hypothesis that a key driver in aesthetic appreciation is the associative thoughts people have in response to the subject matter of an artwork (Vessel and Rubin, 2010).

Drawing on behavioral data, several influential theories of aesthetics have been developed (e.g., Martindale, 1984; Leder et al., 2004; Reber et al., 2004) and considerable progress has been made to identify the visual properties of artworks and the attributes of the viewer that influence aesthetic appreciation (Martindale, 1984; Leder et al., 2004; Reber et al., 2004; Palmer et al., 2013). A key component of all theories of aesthetics is the idea that greater understanding in the viewer reliably enhances aesthetic appreciation (Martindale, 1984; Parsons, 1987; Winston and Cupchik, 1992; Zeki, 1999; Leder et al., 2004; Reber et al., 2004; but see Belke et al., 2015). This is supported by the finding that increased understanding provided by titles reliably enhances the appreciation of photographs (Millis, 2001) and artworks (Russell, 2003; Leder et al., 2006). Greater art expertise also increases the liking of all genres of artwork (Leder et al., 2012) and abstract art in particular (Gordon, 1952; Winston and Cupchik, 1992; Hekkert and van Wieringen, 1996) presumably because art expertise facilitates the understanding of the formal properties of art when representational content is absent (Winston and Cupchik, 1992; Lindell and Mueller, 2011). As Landau et al. (2006) suggest, a lack of understanding in response to abstract art may be the primary reason why naïve adult viewers consistently prefer representational to abstract art (Gordon, 1952; Heinrichs and Cupchik, 1985; Winston and Cupchik, 1992; Mastandrea et al., 2011; see also Leder et al., 2006, 2012).

Although understanding is important to aesthetic appreciation it takes time to develop (Gardner et al., 1975; Parsons, 1987; Belke et al., 2010) and for naïve viewers of art, and children, research suggests that aesthetic appreciation is often quite rudimentary, being governed by a few primary attributes of the artwork, such as subject matter and color (Gordon, 1952; Machotka, 1966; Parsons, 1987; Winston and Cupchik, 1992). In children aesthetic appreciation also depends on their level of neurocognitive development, which will limit the aspects of the artwork and intentions of the artist that they are able to process and understand (see Callaghan and Rochat, 2003). For example, aesthetic appreciation in infants who are too young (<1 year) to understand the representational nature of images is strongly influenced by the color and the visual pattern of the artwork, as measured by their allocation of attention (Cacchione et al., 2011). Once children understand the dual representational nature of pictures (i.e., that a picture is both an object itself and a symbol of something else; DeLoache and Burns, 1994) subject matter becomes more important in aesthetic appreciation (Gordon, 1952; Machotka, 1966). This is mirrored in the development of children’s own drawing ability, which progresses from non-representational scribbling towards the production of more realistic images (e.g., Golomb, 1992, 1994). It is likely, therefore, that older children will be more influenced by the representational content of the artwork (Taunton, 1980) and with the increasing ability to understand the perspectives of others mental states (Theory of Mind, Premack and Woodruff, 1978; Armitage and Allen, 2015) an understanding of the artists’ intentions may also influence aesthetic appreciation.

Leder et al. (2004) proposed that basic forms of aesthetic understanding can involve associative processes, with a viewer generating meaningful information that relates to them because the artwork triggers an association with something they have experienced, remembered, or of which they are aware. The associative ideas of Leder et al. (2004) anticipate the work of Vessel and Rubin (2010) and see also Biederman and Vessel (2006) who proposed that in naïve viewers shared thoughts in response to the content of an artwork have a powerful role in aesthetic appreciation. They reached this conclusion from their finding that the aesthetic appreciation of realistic images (photographs) is more consistent across observers than for abstract images. Vessel and Rubin (2010) propose that, because people have similar experiences in their lives (e.g., when on a beach, walking a dog, or in a car park), when an image represents such a scene it causes similar thoughts in people which influence their liking of the image. Therefore, they explain the greater consistency in representational images as being a product of individuals sharing more thoughts and evaluations for meaningful/realistic images (e.g., a scenic view) than they do for abstract images, causing preferences for the abstract images to be more variable across individuals. In support of Vessel and Rubin’s theory, Schepman et al. (2015b) found, when they measured the valence of participants’ associations in response to abstract and representational artworks, that the valence of the associations correlated with a participant’s liking of an artwork, and both liking and association valence were more consistent across observers for representational than abstract art. Moreover, associations in response to representational art showed greater semantic similarity across participants than associations in response to abstract art (Schepman et al., 2015a).

Vessel and Rubin’s theory suggests that associative thoughts are a major influence in aesthetic evaluations in naïve viewers and determine the consistency of aesthetic preferences across individuals. It can also be expected that over time children will be repeatedly exposed to items in the world that reliably elicit positive experiences (e.g., certain animals, scenes), so that when those items are re-presented as an image they should elicit a positive association. Research suggests that children develop the capacity to understand graphic images as representational symbols towards the end of the third year of life (e.g., Callaghan, 1999), causing pictures to become commonplace in adult-child interactions both at home and in educational settings (e.g., through story book reading; Szechter and Liben, 2004; Danko-McGhee and Slutsky, 2011). If Vessel and Rubin are correct, the association children have developed with the subject matter of a representational picture should influence their appreciation of the picture, and as children will have been exposed to particular items more often as they get older, the strength of the association (and similarity in preference), should also grow stronger with age for representational artworks but not abstract artworks.

Vessel and Rubin’s theory and Schepman et al.’s results indicate an important role for associations in aesthetic appreciation, but very little research has directly examined associations in the development of aesthetic preferences. There is some evidence from unstructured interviews that associations might be important for aesthetic appreciation in young children, with the associations frequently being idiosyncratic but driven by the content (e.g., “giraffe’s back … a dog’s face”, for Picasso’s “Girl Before a Mirror”; Housen, 2002), but the majority of research has emphasized other factors. For example, in Parsons (1987) influential work he interviewed children on their appreciation of artworks and concluded that there were five successive stages of aesthetic understanding, starting from a basic and rudimentary understanding to a more complex and detailed appreciation. In stage 1 color and subject matter were of primary importance in determining a young child’s liking of an artwork (see also Lin and Thomas, 2002) and children in later stages were concerned with how accurately the picture represented the world (stage 2), the expressiveness and emotions elicited by the artwork (stage 3), and the medium, style, formal properties, organization and artist’s intentions (stage 4). However, associations were not explicitly listed by Parsons as having a particularly powerful influence on aesthetic appreciation in any of the five stages. Despite this, Parsons observed that some content resulted in a high level of agreement in aesthetic evaluation, particularly for children in stage 1, with nearly all the children liking a particular artwork because it featured a dog. Similarly DeSantis and Housen (1996) and Danko-McGhee and Slutsky (2011) found that children liked certain artworks because of the content (e.g., fish, dogs, etc.), which they describe as a process of “simple association”, and Freeman and Sanger (1995) found that the majority of children thought a picture would be bad if it depicted something that was ugly. These findings support the view that aesthetic preferences for representational art can be remarkably consistent if the thoughts and emotions elicited by the artwork’s content are relatively similar across individuals.

Parson’s work was developed further by Lin and Thomas (2002) who examined aesthetic preferences in children (4–5 years, 7–9 years, 12–14 years), and adults (non-art undergraduate students, art and design students), for artworks from five genres (abstract, fine, contemporary, humorous, and cartoon). For each genre participants were presented with five artworks and were asked to select the artwork they most liked, and then describe what the picture was about and why they liked it. Lin and Thomas then classified the participant’s statements into nine categories (e.g., subject matter, color, form, association etc.) to examine how frequently different types of justification were mentioned by participants when explaining their choice. They concluded from their findings that the development of aesthetic understanding did not follow the strict sequence of stages that Parsons originally proposed, but could branch in different directions and that understanding and preferences changed with age. Only art students showed different reactions for the different genres of art, showing that naïve viewers have relatively similar responses irrespective of the genre and that exposure to, and knowledge of art, is crucial to the nature of the aesthetic experience and the depth of processing achieved (Winston and Cupchik, 1992; Augustin and Leder, 2006; Leder, 2013). In agreement with other studies subject matter and color were frequently mentioned and were of primary importance for aesthetic appreciation in all groups (Machotka, 1966; Rump and Southgate, 1967; Rosentiel et al., 1978; Bell and Bell, 1979), though color was very important for the youngest group and declined somewhat with increased age. Finally, despite having an “association” coding category, few participants referred to associations when explaining their preferences.

It is possible, however, that the infrequent reference to associations was due to Lin and Thomas’ procedure of requiring participants to select a particular artwork and then explain their justification. If a few attributes determine liking in most instances (e.g., subject matter, color, artistic properties) then most of the artworks will have been chosen on the basis of these attributes (and will also have been the participant’s justification for their choice). Therefore, asking participants to select the artwork might have limited the complexity and range of participant’s justifications for their aesthetic preferences, and prevented the detection of less salient influences on choice. We aimed to overcome this limitation by asking participants to explain their level of liking for an artwork without the artwork being pre-selected by the participant.

It is also apparent from the literature that the extent to which responses to artworks are viewed as associations varies markedly but is crucial to whether findings are interpreted as supporting Vessel and Rubin’s theory. It is possible that Lin and Thomas (2002) did not find a large role for associations because they used a specific definition, of “being reminded of material”. If a broader concept of associations is adopted to refer to interpretive thoughts (with a valence) generated by the content of an artwork then it appears that those thoughts may determine liking in naïve viewers (Parsons, 1987; Freeman and Sanger, 1995; DeSantis and Housen, 1996). It is this concept of associations that we subscribe to and which we believe might cause the earlier development of shared preferences for representational art than abstract art. It also corresponds to Vessel and Rubin’s (2010) description of semantic associations, which are an interpretation of the image’s content, and are distinct from other low-level perceptual associations that can be elicited by the physical features of an the image. An example of a semantic association would be thoughts of a “holiday” elicited by the depiction of a beach, or “shopping/work” from an image of a car park, while a perceptual association might be thoughts of “hard” or “cold” in response to the particular lines or colors of an image. It is important to note that a semantic association is not a simple reiteration of the subject matter but has a level of interpretation of the image, and which Vessel and Rubin suggest are elicited much more readily by representational images than abstract images.

The development of aesthetic preferences towards different styles of artwork was also examined very recently by Schabmann et al. (2015) in a study whose publication occurred when our own empirical work was already complete. They asked children (age 4–6 and 9–11) to rate different styles of artworks on four dimensions, namely liking, valence, understanding, arousal and categorized the children’s verbal responses to questions about their evaluation of the artwork. They found that emotion was an important determinant of liking at all ages, but particularly for the younger children, with older children’s evaluations becoming increasingly cognitively based.

Our study aimed to test, in primary school children (aged 4, 6, 8 and 10) predictions derived from Vessel and Rubin’s (2010) theory of shared aesthetic preferences, whilst also building on the work of Lin and Thomas (2002). We gathered liking data using a quantitative liking scale, in addition to recording children’s verbal justifications of their aesthetic evaluations. This represented a balanced approach, in which all children could communicate their evaluations relatively independently of language ability, as well as providing their own reasoning for these evaluations which would not be revealed through a forced choice task.

Of focal interest to the association theory, our first hypothesis was that shared liking would emerge more strongly at older ages for representational as opposed to abstract artworks, strengthening the view that thoughts triggered by the subject matter are an important driver in aesthetic appreciation. Based on the findings of previous research (Trautner, 2008), our second hypothesis was that a preference for representational art would emerge at older ages, potentially because older children are increasingly influenced by the representational content of the artworks in the same way that adults are (e.g., Landau et al., 2006). In relation to the justifications provided, it was expected that a greater range and complexity of reasons would emerge with advancing age, due to a more sophisticated understanding of art underpinned by neurocognitive development, which, in turn, may drive aesthetic development (hypothesis 3).

We treated the justification data on an exploratory basis, and thus without formal hypotheses. We explored whether subject matter, associations, understanding and interpretation, and mood and emotions played a stronger role in the justifications for representational than abstract artwork. Based on prior work, we also explored whether color and subject matter would be mentioned particularly frequently in the justifications for the aesthetic ratings (e.g., Gardner et al., 1975; Parsons, 1987; Lin and Thomas, 2002). We also aimed to explore whether color and other formal artistic properties would be more prominent in justifications for abstract than for representational art. We made a number of further observations which we linked to aesthetic and general neurocognitive development. These are reported in the “Results” Section.

## Materials and Methods

### Participants

Eighty children from a National Curriculum Primary school in Cheshire participated in the study. The school’s achievement levels were approximately average (1% below the national mean) for the year in which testing took place. Ethical approval was received from the Ethics Committee of the Department of Psychology, University of Chester, and complied with British Psychological Society ethical guidelines. The school head teacher gave provisional consent for the study to take place and participants were then recruited from classrooms teaching 4-, 6-, 8- and 10-year-old children via parental opt-out consent. Of those available to take part in the study, the class teacher selected 20 children from each age group. Teachers were instructed not to choose children who had particular experience or interest in the visual arts. All children verbally assented to take part and none refused to participate. The characteristics of the sample were as follows: 4-year-olds (mean age 4.7, *SD* 3 months; 15 males and 5 females); 6-year-olds (mean age 6.4, *SD* 4 months; 8 males, 12 females); 8-year-olds (mean age 8.7, *SD* 3 months; 10 males, 10 females); 10-year-olds (mean age 10.6; *SD* 4 months; 10 males, 10 females).

### Materials

An initial set of 40 artworks (20 representational and 20 abstract) was selected by authors PR, AS and JK, following image searches on the internet. This initial set was developed from the set used in Schepman et al. (2015a, Experiment 1), with the replacement of some abstract artworks, with an aim of enhancing the variation in responses in the abstract set. The artworks selected were not famous to reduce the risk that viewers had already seen the work and had a pre-formed opinion. We included in our classification of representational artworks those that depicted real-life entities without gross distortions in shape or color. Abstract artworks could not contain any recognizable objects or scenes, but we did include artworks which contained recognizable shapes.

Undergraduate student raters provided pre-test ratings on a seven-point scale of the artworks’ attractiveness, colorfulness, interest, liking, and negativity/positivity (see Schepman et al., 2015b for fuller details of the procedure, which matches the current procedure). Based on this pre-test, we selected 20 artworks, 10 representational and 10 abstract (see Appendix 1), as we felt that would be the maximum that very young children would be able to work with. This is consistent with the number of artworks presented to children in previous studies including Machotka (1966) and Lin and Thomas (2002). In our selection, we ensured that any differences in ratings in the larger set of 40 artworks were also present in the selection. We present the descriptive statistical properties of the selected 20 artworks (10 per art type) in Table 1, based on ratings by 23 student raters.

**Table 1:** **Pre-test rating means and standard deviations given by undergraduate students to the artworks used in the main study, by art type, using a 1–7 scale**.

	Abstract		Representational	
	Mean	*SD*	Mean	*SD*
Attractive	3.66	0.74	4.18	1.06
Colorful	4.71	1.42	4.54	1.15
Interest	3.82	0.77	4.31	0.66
Liking	3.85	0.67	4.59	0.83
Negativity/Positivity	3.99	0.83	4.89	1.28

All artworks were reproduced in high quality color print on white A4 paper and were presented in a booklet. Three versions of this booklet were prepared with three different randomized orders of the artworks that were counterbalanced across participants to prevent any order effects. Alongside the booklet, participants were presented with a rating sheet. On the rating sheet pictures of 1, 2, 3, 4, and 5 stars were shown labeled with the corresponding number. Star charts are commonly used as part of educational reward systems (e.g., Kazdin, 2001) and the idea of more stars representing greater liking would be familiar to participants, helping to locate their understanding of the task within a familiar and concrete context. Liking is also “the dimension that best captures the aesthetic response” (Schabmann et al., 2015), and the star rating may have ameliorated some of the difficulties reported by Schabmann et al. (2015) when testing kindergarten children with a nine point Likert scale.

### Procedure

All participants were tested individually by author JK at a desk in a quiet room next to their usual classroom.

The study was introduced to each child as follows: “*We see pictures every day, for example in books and on walls at school and at home. We may like some pictures more than we like others. Today I am interested in what pictures you like. There is no right or wrong answer; I just want you to tell me what you think of each picture that I show you*.” Participants were also instructed that they could ask for a break at any time during the procedure. Each artwork in the booklet was presented sequentially to the participants with the following instruction repeated for each of the 20 artworks *“I would like to know how much you like this picture. Would you give it 1 star (you don’t like the picture at all), 2 stars (you think the picture is ok but that are some parts that you don’t like), 3 stars (the picture is good. You like it), 4 stars (the picture is very good. You like it a lot) or 5 stars? (The picture is excellent. You love it).”* Participants were then instructed to point to the number of stars that they wanted to give the artwork on the star rating sheet. If necessary the instructions were repeated.

After indicating their rating for each individual artwork participants were then asked “why did you give that picture 1–5 stars?” The instruction was repeated as necessary to elicit a response and any queries raised by the participants were answered as follows: *“I am interested to know the reasons why you gave this picture 1–5 stars. There is no right or wrong answer. I only want to know what you think about the picture.”* Due to the potentially limited verbal abilities of some of the children, three additional categories of prompts were used by JK to support and clarify participant’s responses. Firstly, for basic responses without any explanation (for e.g., “I like it”) participants were prompted by asking “why?” or “what?” questions to elicit further detail. Secondly, if participants were explaining a concept but were unable to retrieve the appropriate word to describe it (or used the incorrect word), then JK provided the correct word (e.g., “calf”). Finally, if participants provided an explanation with reference to part of a picture but it was unclear what part they were referring to, JK asked participants to clarify this.

To keep the time frame of the study manageable for primary school children each of the 20 artworks was presented to the participant for a maximum of 5 min. All justifications were audio recorded and transcribed for analysis. It is possible that participants may have been able to give more detailed responses with additional questioning from JK. However this may have increased the possibility of interviewer bias. Limiting the questions posed about the artwork also ensured that the justifications given were the most salient to the participants within the time allocated to each picture.

## Results

### Rating Data

#### Cross-Rater Agreement as a Function of Art Type and Age Group

**Figure 1:**
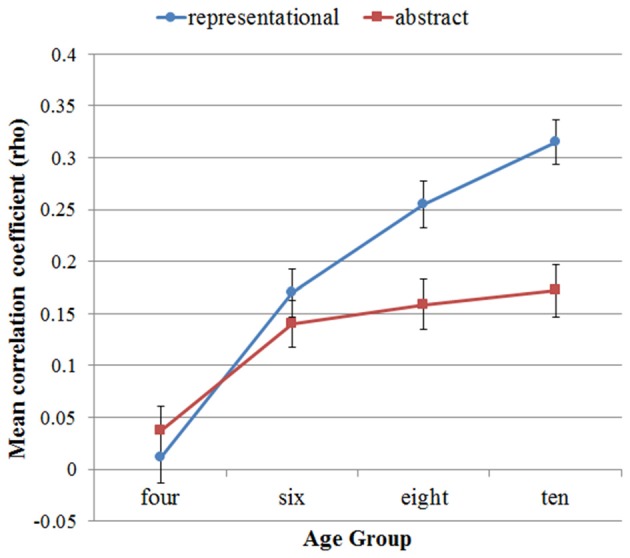
**Mean correlation coefficients expressing the level of cross-rater convergence in star ratings, by age group and art type, with standard error of the means indicated as bars**.

In relation to our first hypothesis, that convergence of ratings across individuals may be greater for representational than abstract art, derived from Vessel and Rubin (2010) and building on Schepman et al. (2015b), we report the convergence of ratings as a function of art type and age group first. Following the method used in Schepman et al. (2015b), participants’ star ratings were analyzed for cross-rater agreement using non-parametric Spearman’s rank correlation coefficients, in which each participant’s rating for each artwork was correlated with the ratings given by all other participants. These correlation matrices were generated separately for each age group and art type. The sets of correlation coefficients thus obtained were compared for differences between art types in each age group, using non-parametric pairwise comparisons, namely Wilcoxon signed-rank tests, to test whether cross-rater agreement differed as a function of art type in the different age groups. Mean correlation coefficients as a function of age group and art type are shown in Figure 1.

From these data, it is clear that the children at age 4 showed very low inter-rater agreement for both abstract (mean rho = 0.04, *SD* = 0.33) and representational art (mean rho = 0.01, *SD* = 0.33), and their agreement did not differ significantly as a function of art type (*Z* = −0.88, *p* = 0.38). At age 6, the mean correlation coefficients were somewhat higher (mean rho = 0.14, *SD* = 0.31 for abstract; mean rho = 0.17, *SD* = 0.32 for representational art), but they did not differ significantly as a function of art type *Z* = −1.11, *p* = 0.27). The agreement for representational art was markedly higher at age 8 (mean rho = 0.26, *SD* = 0.31) and age 10 (mean rho = 0.32; *SD* = 0.30), and the agreement for abstract somewhat higher than in the younger age groups at age 8 (mean rho = 0.16, *SD* = 0.33) and age 10 (mean rho = 0.17, *SD* = 0.35). Importantly, at ages 8 and 10 there were significant differences in agreement as a function of art type, with children agreeing significantly more with their peers on their ratings of representational artwork than abstract artwork (at age 8, *Z* = −2.89, *p* = 0.004; at age 10, *Z* = −3.75, *p* < 0.001). Thus, based on these data, it seems that a higher level of convergence for representational artwork than for abstract artworks can be observed from age 8, and it continues to be observed at age 10. This supports our first hypothesis.

Not focally related to our hypotheses, but at the request of a reviewer and of likely help with the interpretation of the overall pattern, we also report the effects of age on the correlations. As main effects, the correlations increased significantly with age for both abstract (Friedman $X_{\left(3\right)}^{2}$ = 18.22, *p* < 0.001) and representational artworks (Friedman $X_{\left(3\right)}^{2}$ = 68.70, *p* < 0.001). Further pairwise Wilcoxon tests showed that for abstract art, the correlations only increased significantly when comparing age 4 to age 6 (*Z* = −3.198, *p* = 0.001), but not for the other adjacent age pairs (*Z* > −0.5, *p* > 0.6), while for representational art, convergence rose between ages 4 and 6 (*Z* = −4.385, *p* < 0.001), 6 and 8 (*Z* = −2.385, *p* = 0.017), but the rise only approached significance between ages 8 and 10 (*Z* = −1.764, *p* = 0.078).

#### Ratings by Art Type and Age Group

To test hypothesis 2 and to gain insight into age trends in overall liking of abstract and representational art, star ratings were averaged for each child and each art type. One data point from one participant was missing as it had not been collected, but this was estimated using the mean of the condition for that child. Resulting means and standard deviations for each age group and condition are presented in Table 2. At the request of a reviewer, we report the inferential statistics against an alpha of 0.05/4 = 0.0125, by way of Bonferroni correction for multiple comparisons, while reporting the original *p*-values yielded by the inferential tests in Table 2. These analyses showed some preference trends, which appeared to show a stronger liking for abstract art at age 6, and a stronger liking for representational art at age 10, but these were not significant against the corrected alpha. Thus, the data did not provide support for hypothesis 2.

**Table 2:** **Means and *SD*s for ratings given by children in the four age groups on a scale of 1–5 stars as a function of art type, with *Z* and *p* yielded by a Wilcoxon test for each pairwise comparison in the rightmost columns**.

	Abstract		Representational	
Age group	Mean	*SD*	Mean	*SD*	*Z*	*p* (α = 0.0125)
4	3.37	0.72	3.26	0.72	−0.24	0.81
6	3.7	0.55	3.26	0.7	−2.48	0.013
8	3.47	0.67	3.49	0.58	−0.2	0.844
10	3.3	0.67	3.76	0.58	−2.2	0.028

*Note that the Bonferroni-corrected alpha for this analysis was 0.0125*.

### Justifications

#### Transcription

Two independent raters (authors JL and AL) transcribed participants’ verbal responses for each artwork. Each rater transcribed the verbal responses of half of the participants within each age group. Transcription was verbatim and the raters were blind to the age of the participants.

#### Development and Refinement of the Coding Scheme

To classify and quantify participants’ verbal justifications for their preferences an initial coding scheme was developed by JK, PR and AS which included 16 of the most prominently used categories drawn from a review of relevant literature (e.g., Machotka, 1966; Parsons, 1987; Lin and Thomas, 2002) as well as from JK’s direct experience of listening to the participants responses.

The initial categories were discussed individually with each coder (authors JL and AL), and a booklet was produced defining and explaining the categories as well as giving illustrative examples of potential quotes which could exemplify these. A sample of ten participants was randomly selected from the data set and each rater was instructed to individually code this set of responses using the initial coding scheme.

Following the initial coding, all authors met to discuss and evaluate the level of agreement within the provisional sample and to discuss the coders’ understanding and use of the coding scheme. Following this discussion, to achieve the most parsimonious scheme, two categories were deleted because they overlapped with other criteria and thus did not represent a unique basis for justifications. The final simplified coding scheme included the 14 categories which are outlined in Table 3. It is to be particularly noted that we had to demarcate associations from subject matter in a precise way to ensure coding reliability.

**Table 3:** **Final 14 coding categories with brief descriptions and a sample quote for each category**.

Category	Description
Formal artistic properties	Any reference to the formal artistic properties of the artwork such as line, composition or style. “I don’t really like how it’s set out.”
Color	Any reference to the colors used in the artwork. This could be simple naming or counting of colors (or any reference to color as a means to create, form or express in the artwork). “Because I like all the yellow bits over there.”
Artist	Any reference to the person who created the artwork either directly or indirectly. This could include reference to the artist as an intentional creator of the artwork or to the technical skill, ability or proficiency of the artist. “I like how they have made the water go back and make the shadows of the dog and not just a squiggle.”
Subject matter	Any simple reference to, or statement of, the content or subject matter of the artwork including objects, events or activities that are formally represented. “Because it’s got fish in it. I don’t like fish.”
Associations	Any justification where a connection or link is made between the artwork and the participants own personal life, experience or memories. “Because it reminds me of one of my friends. It actually looks really like her. She giggles a lot and is laughing all the time.”
Understanding/Interpretation	Any reference to comprehending (or lack of comprehension) of the artwork or any aspects of it, or any attempt to try to interpret the meaning of the artwork or to build an explanatory narrative for it. “I don’t know what it’s supposed to be”.
Mood/Emotion	Any reference to feeling, state of mind or prevailing tone of the artwork and its subject matter, or relating to the viewer or artist. “Because she looks happy.”
Interest	Any reference to basic interest in the artwork, or the artwork commanding attention or attracting curiosity. “It looks quite interesting.”
Function	Any suggestion on the practical usage of the artwork. “Well, I would see it on display but not at an art gallery.”
Comparison	Any preferences which are justified through comparison (for example, to other artworks in the stimuli set, or to previous scores given by the participant). “It’s good but it’s not the best of all the drawings.”
History/Culture	Any preferences which are explained or justified by relating the artwork or anything in it to culture or history. “That’s like in the building where the earthquake struck Kefalonia.”
Perceptual fluency	Any reference to the ease, difficulty or speed at which the information in the artwork can be processed. “You can see what it is at a glance.”
Basic liking	Any basic reference to liking or disliking the picture without elaboration or reference to any other theme. “Because I like it.”
Other	Any preferences given which are not accounted for by the above themes. “I don’t have a reason I just think it’s three.”

#### Coding

Authors JL and AL used the 14-item coding scheme to classify participants’ verbal justifications for their ratings of the 20 artworks. They initially coded the justifications that they had previously transcribed as this ensured a high degree of familiarity with the data. They coded for the presence vs. absence of the categories for each child’s response to each artwork, but not the number of times that a particular category arose within each individual justification. Participants’ verbal justifications could fall within more than one category, and all categories that were relevant for that individual justification were documented. The category “Basic Liking” was only used if no other reasons were given, and the category “Other” was used only when none of the other categories applied. Following initial coding of half the data, coders blindly coded the other half of the sample, and agreement was checked. If the two coders had a disagreement, they jointly revisited the conflicting code and reached agreement by discussion. In some instances, the coding disagreements were simple entry errors, and these were repaired. Analysis of all 1600 justifications showed that in 92.9% of the justifications, the raters agreed on all 14 codes chosen, while in 7.1% of justifications, they needed to discuss one or more codes to reach agreement on the overall coding of the justification. This was a good level of agreement, indicating that the coding scheme was usable, reliable and valid.

#### Number of Categories used in Justifications

As children age and language and cognition develop, it is conceivable that they are able to provide more complex, multi-faceted justifications for their ratings of artworks. To see whether, as predicted by hypothesis 3, this was the case in our data, we calculated the mean number of justifications provided per child, and subjected this to a Kruskal-Wallis test with age group as the independent variable. This showed that the number of categories rose as a function of age, with 4-year-olds providing 1.2 categories on average, 6-year-olds 1.4, and both 8-year-olds and 10-year-olds 2.0. This increase was significant, $X_{\left(3\right)}^{2}$ = 42.895, *p* < 0.001. To examine where the increase differed significantly between adjacent age groups, follow-up Mann-Whitney tests were run, and these showed that the increment between ages 6 and 8 was the only significant contrast, *Z* = −4.224, *p* < 0.001. This suggests a possible step-change in the complexity of the justifications between ages 6 and 8. This supports hypothesis 3, and indicates the ages across which the complexity increases most.

#### Frequency of Coding Categories

**Figure 2:**
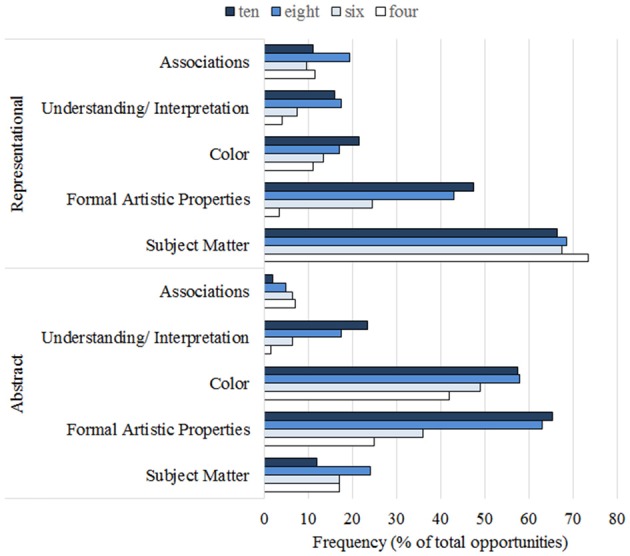
**Frequency of occurrence (in percentages of the total number of opportunities) of the five most prominent coding categories; separated for abstract and representational art, with 4, 6, 8 and 10 referring to age groups**.

As observed, children used a higher number of codes in older age groups. To further examine how the different categories were used by the children across the two types of artwork as a function of age, the mean frequency of usage of the 14 coding categories was calculated as a percentage of the total of the number of opportunities for each child and art type. The resulting means can be seen in Table 4, with visualization of the most common codes in Figure 2.

**Table 4:** **Frequency of occurrence (in percentages of the total number of opportunities) of the 14 coding categories; separated for abstract and representational art, with A = Abstract, R = Representational, and 4, 6, 8 and 10 referring to age groups**.

	A4	R4	A6	R6	A8	R8	A10	R10	Overall
Formal artistic properties	25	3.5	36	24.5	63	43	65.5	47.5	38.5
Color	42	11	49	13.5	58	17	57.5	21.5	33.7
Artist	0	0	2.5	1.5	12	9.5	13	11	6.2
Subject matter	17	73.5	17	67.5	24	68.5	12	66.5	43.3
Associations	7	11.5	6.5	9.5	5	19.5	2	11	9
Understanding/Interpretation	1.5	4	6.5	7.5	17.5	17.5	23.5	16	11.8
Mood emotion	1.5	4.5	2	4	1	5	0.5	13	4
Interest	0	0.5	2.5	4	4.5	5.5	10.5	7.5	4.4
Function	0	0	0	0	0	0	1.5	0	0.2
Comparison	0	0	0	0	8	3	4	6	2.7
History culture	0	2	0	4.5	0	4	0	2	1.6
Perceptual fluency	0	0	0	0	0	0	4	2.5	0.8
Basic liking	13.5	11.5	4	5	2	1	0	0.5	4.7
Other	7	4.5	2	3	1	1.5	0.5	0	2.5

*Overall mean frequencies collapsing over age groups and art type are in the final column*.

The most frequent codes, which were used at a frequency of 10% or greater collapsing over age groups and art type, were formal artistic properties, color, subject matter, and understanding/interpretation. The other codes were used more sparingly by the children, though associations reached 9% overall.

#### Overall Age Effects on Frequency of Usage of Coding Categories

Using Kruskal-Wallis tests with the frequency of usage of a coding category per child as the dependent variable and age group as the independent variable, it was found that there were significant effects of age on the use of some codes, namely, formal artistic properties, $X_{\left(3\right)}^{2}$ = 38.3, *p* < 0.001, with an increasing use with age, which was significant on a Mann-Whitney comparison between ages 4 and 6 (*Z* = −3.304, *p* = 0.001, and ages 6 and 8, *Z* = −2.936, *p* = 0.003) but not ages 8 and 10. Further, reference to the artist, $X_{\left(3\right)}^{2}$ = 25.7, *p* < 0.001, also increased, with two significant increments between ages 4 and 6, *Z* = −2.080, *p* = 0.038, and 6 and 8, *Z* = −2.687, *p* = 0.017, but not 8 and 10. Understanding and interpretation, $X_{\left(3\right)}^{2}$ = 26.3, *p* < 0.001, also increased with age, with the age 6 to age 8 increment being the only significant one, *Z* = −2.769, *p* = 0.006. Comparison also showed an age effect, $X_{\left(3\right)}^{2}$ = 26.2, *p* < 0.001, with a sharp increase between ages 6 and 8, again the only significant increment in usage, *Z* = −3.789, *p* < 0.001. Perceptual fluency, $X_{\left(3\right)}^{2}$ = 15.78, *p* = 0.001, showed no usage at all for ages 4–8, with a sudden onset of usage at age 10, which was significant, *Z* = −2.354, *p* = 0.019.

Some categories showed downward trends. Usage of basic liking dropped significantly across the age groups, $X_{\left(3\right)}^{2}$ = 13.35, *p* = 0.004, with the most pronounced drop occurring between ages 4 and 6, though this contrast did not quite reach significance on a pairwise Mann-Whitney comparison. “Other” showed a similar numerical trend, but this was not significant. As these categories were used by the coders when no other labels applied, this is likely to be due to the use of other labels.

#### Justification Category Usage in Abstract vs. Representational Artwork

**Figure 3:**
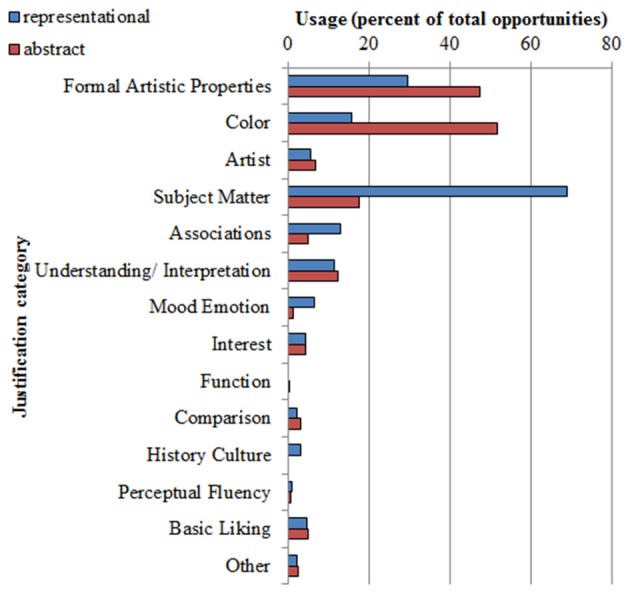
**Mean use of the justification categories by art type, collapsing across age groups, expressed as a percentage of the total number of opportunities**.

The data showed that the use of categories varied by art type. The mean percentage use for the two art types, collapsing across the age groups, are shown in Figure 3. A series of Wilcoxon signed rank tests was run to test whether the main effect of art type on usage of each category was significant. In four categories, justifications were used significantly more frequently as a justification of a rating of representational than abstract artworks. These were subject matter, *Z* = 7.659, *p* < 0.001; associations, *Z* = −4.572, *p* < 0.001; mood and emotion, *Z* = 4.462, *p* < 0.001; and history and culture, *Z* = 3.530, *p* < 0.001. Two patterns differed in the opposite direction. It was found that justifications featuring color were significantly more common in abstract than representational artwork, *Z* = 7.369, *p* < 0.001, as were justifications featuring formal artistic properties, *Z* = −4.951, *p* < 0.001. No other contrasts reached or approached significance.

Because there is no standard statistical test for non-parametric data that is equivalent to the factorial interaction in ANOVA, we tested for interactions by entering all trials separately (not averaged by subject), and running Chi-Square tests, with Age Group and Art Type as factors. These showed only one significant interaction pattern, namely for formal artistic properties, $X_{\left(3\right)}^{2}$ = 18.333, *p* < 0.001. Further analysis of this main effect using four Wilcoxon tests (one for each age group) showed that the difference between abstract and representational artwork was significant in all age groups, except at age 10, where the difference in the same direction narrowly missed significance. In all other categories, the Chi-Square tests did not reach significance. This confirms the patterns that can also be seen in the means, that the effect of art type on category usage was stable across the age groups.

## Discussion

A number of important results were obtained that are central to furthering the understanding of the development of children’s aesthetic appreciation. The most noteworthy was a greater shared liking for representational artworks compared to abstract artworks at age 8 and 10. This finding was in line with our primary hypothesis and will be discussed shortly. We also observed a wider range of aesthetic justifications than previously identified and a general increase of justifications with age. Finally, we observed a clear pattern of different justifications as a function of art type which was stable across the age groups.

### Convergence as a Function of Art Type and Age

The result central to the aims of this research is the finding that significantly greater shared liking was present at ages 8 and 10 for representational art compared to abstract art, supporting our first and primary hypothesis. This finding was predicted from Vessel and Rubin’s theory and as noted previously, can be explained in terms of older children having developed similar thoughts in response to the artwork’s subject matter and which influenced their liking in systematic and predictable ways. We are not aware of an alternative theory of aesthetics that would have predicted, or could explain, this finding. The lack of convergence in preference for representational artworks in younger children is in agreement with the view that it takes time to develop preferences and associations in relation to the depicted subject matter.

However, there are alternative possible explanations for the lack of or lower level of convergence with abstract art that we must consider. At a young age, evaluations of art may not yet be stable, in that children might not give the same responses from test to re-test (Pugach et al., 2014). Pugach et al. (2014) found that younger children (3–6 years) show less stability in aesthetic judgment than older children (7–9 years), meaning that individual ratings may be more “random” in a younger child compared to an older child. Another possibility is that young children simply do not know how to use rating scales. An inspection of the data suggests that it is possible that at age 4 such an explanation may hold, because convergence was very low for both art types at that age. However, convergence increases for both abstract and representational art between ages 4 and 6, and then continues to rise for only representational art between ages 6 and 8, with a further non-significant trend between ages 8 and 10. This difference in the rise in convergence as a function of art type is not compatible with an explanation in which there is a general inability to provide stable judgments or to use a rating scale, as under such an explanation, convergence should not increase for either type of artwork. Thus, the difference in convergence between abstract and representational art at ages 8 and 10 seems more likely to be evidence of a shared liking via meanings and associations for representational artworks only, in line with Vessel and Rubin (2010) and Schepman et al. (2015b) than for a generalized instability in using the ratings, particularly from age 6 upwards.

A potentially profound implication of this finding is that similar patterns of shared liking can be expected for the development of children’s preferences toward other items, such as toys, consumer items and foods. These preferences may show a different developmental trajectory, depending on the nature of the item, consistency of exposure and strength of emotional response, but, based on these results we predict that the development of convergence in liking is a general (and measurable) phenomenon. In addition, if Vessel and Rubin’s (2010) theory is correct then it might account for the development of shared liking in a wide range of circumstances. For instance, if repeated exposure to items in the world elicits similar experiences in people, which comprise similar thoughts and emotions, then this may be an important way in which children develop shared meanings and values about the world. A potential mechanism by which associations between entities in the real world and affective interpretations of those entities are formed could include statistical learning (Saffran et al., 1996; Kushnir et al., 2010). We will return to other predictions from this theory when we consider the verbal justifications, shortly.

### Art Type Preference

We note that in our second hypothesis we expected ratings for representational art to be higher than those for abstract art in the older age group, but we did not obtain strong statistical evidence for this. It is possible that this analysis suffered from a lack of statistical power. Compared to the adult data reported in Table 1 and in Schepman et al. (2015b) we used a narrower rating scale (1–5 stars for the children, 1–7 points for the adults). This was based on our prior evaluation that children would be more able to cope with a five-star rating system than one which had seven points, in part because children may have used such star ratings in their daily lives (e.g., on reviews for books or games etc.). The narrower scale may have reduced measurement sensitivity and statistical power in this part of the data set. We exercise caution in evaluating hypothesis 2 due to this issue, which makes it difficult to distinguish between a genuine null effect and a type II error.

### Verbal Justifications

The three most common categories used to justify ratings were formal artistic properties, color, and subject matter, which are the same as those identified by Lin and Thomas (2002; note that their term for our formal artistic properties is “medium”). Additional justifications also influenced the children’s preferences, which were not detected to a significant extent by Lin and Thomas (2002), and included understanding and interpretation, associations, and references to the artist (11.8, 9 and 6.2% of usage frequency, respectively). Therefore our methodology appears to have been able to identify the most important categories whilst also being sensitive at detecting nuances in the multi-faceted nature of aesthetic justifications that have not been observed before. To a substantial degree the properties of the artwork determined the justifications the children gave for liking an artwork, so that there were systematic and predictable differences between the types of art (see also Lin and Thomas, 2002). There were also clear effects of age. The remainder of the findings will be discussed in two subsections, namely the effect of art type on frequency of use of the justification categories, and the effect of age on the number and type of justification categories used.

### Usage of the Categories as a Function of Art Type

Representational artworks appear to be liked because of what they depict. Subject matter, associations, mood and emotion, and history and culture were used more frequently as justifications for evaluations of representational than abstract artworks. Although associations were provided as justifications significantly more for representational than for abstract artworks, they were not common, occurring with an overall frequency of 12.9% for representational art. This is a much higher frequency than obtained by Lin and Thomas (2002), perhaps because our definition was slightly broader than theirs, but it is lower than would be expected if associations have an important influence on liking and shared preferences.

The apparently limited incidence of associative thoughts is likely to be a product of how we defined the category of associations to capture memories and personal experiences, whilst being distinct from the category of subject matter, which required its own category to ensure coding reliability. As explained in the results section this was needed to implement a reliable coding scheme that made a distinction between aspects of associative thoughts and subject matter. The data show that for representational art subject matter is overwhelmingly the most frequent justification mentioned (69%), showing that thoughts in response to the subject matter determined liking more than any other aspect of the representational artwork. It is probable, therefore, that some justifications that were based on semantic interpretations of the artwork’s content will have been categorized as subject matter rather than associations. For example, in response to artwork 5 (5: Mark Peterson: ‘55 T-Bird), which depicts a car on a beach, a child gave the artwork one star “Cos there’s a car on it”. This does not explicitly reveal memories or personal experiences and was therefore not coded as showing associations, but nevertheless indicates that the subject matter was interpreted negatively and this drove the rating. Similarly, for artwork 6 (Jay Kemp: Return to Sender), which depicts a dog swimming with a stick in its mouth, one child gave the justification “I like it cos the dog’s fetching the thing”, clearly illustrating an interpretation beyond simply naming the most salient object. Instead, the responses reveal more complex interpretative processes, with different viewers selecting different aspects of the subject matter in their justifications. Such interpretations were also observed by Schepman et al. (2015a) with adult participants. While these examples were not coded as an association under our definition, they are in agreement with the idea that interpretive thoughts elicited by the subject matter determine liking (e.g., Leder et al., 2004; Vessel and Rubin, 2010; Schepman et al., 2015b). Therefore, while the infrequent use of associations as a justification category appears to cast doubt on the suggested role of associative thoughts in liking, we believe this is an unavoidable consequence of the need for our coding scheme to be reliable. This very interesting issue could be further explored in future studies by the use of a more quantitative analysis of semantic associations, for example using the method devised by Schepman et al. (2015a).

Abstract artworks appear to be liked for their colors and formal artistic properties, as these were more frequently used as justifications for abstract than representational work. It is apparent, therefore, that even young children were sensitive to the visual properties of the art and that these seemed to play more of a role in the evaluation of abstract than representational art, perhaps because, in the absence of discernible subject matter, color and formal properties such as line and composition are more salient (Jolley and Thomas, 1994). This result echoes observations by Gardner et al. (1975), who noted that 4- and 5-year-olds liked abstract art because of the “pretty colors” and “nice design”. Interestingly, we found some evidence of children attempting to impose meanings or associations on abstract art, which was also observed by Gardner et al. (1975). Such “romancing” can also be seen in the early stages of children’s own drawing productions where representational intentions may frequently change (Golomb, 1982, 1992).

As evidenced in Schepman et al. (2015a), in adult viewers there appears to be some shared meaning in response to abstract works (see also Vessel and Rubin, 2010). The responses made by the children in the current study also showed some overlap alongside the hypothesized idiosyncrasy in interpretation. For example, in response to artwork 15 (Stephanie Kordan Dardashti: Desire Red) one child’s justification was “I like the leaves”, and a further child offered “… the leaves are covering the middle …”, another “… it’s like fire”, and another “… like an Indian campfire …”, while another stated “… like a city”. This overlap in meaning (leaves, fire), with some idiosyncrasy (city) and a larger number of responses not related to subject matter at all illustrates the pattern found more widely in the current data. The question to what extent any semantic interpretations of abstract art are shared across children seems an interesting line to investigate in more detail in future research, because it may provide an insight into children’s interpretation of visual language.

### Effects of Age on Justification Categories

As predicted in hypothesis 3, an age-related increase in the variety and complexity of justifications was found (on average 1.2 categories were used at 4 years, 1.4 at 6 years, and 2 categories at 8 and 10 years), which a significant increase from ages 6–8. Moreover, there was no loss of justifications across the ages, but a general expansion of the categories, with an increased role for cognitive processes (see also Schabmann et al., 2015). The notable increase in the complexity of justifications from 6–8 suggests a rapid development in the number of factors that influence aesthetic appreciation across this age boundary. A developmental explanation is offered by Machotka (1966), who reported similar results and suggested that this is analogous to the transition between pre-operational and concrete operational thought in Piaget’s (1947) theory of intellectual development. Undoubtedly part of the increase in the complexity of the justifications with age is due to neurocognitive development, with basic sensori-motor areas of the brain maturing earlier than higher-order association areas that support more integrative functions (see Weisner, 1996; Gogtay et al., 2004; Jambon and Smetana, 2014; for a review see Del Giudice, 2014). Brain maturation also underpins the further development of cognitive processes such as working memory (Bunge and Wright, 2007), metacognition, and Theory of Mind, which may be necessary for children to be able to provide some justifications. Finally we must note that a general increase of language ability may also play a role in the increase of the number of categories with age, particularly when examining changes from ages 4–6, where we saw a significant drop in basic liking in favor of more specific justifications that may be harder for children with limited language abilities to articulate.

One key category that increased significantly in frequency from ages 6–8 was understanding and interpretation. Schabmann et al. (2015) also showed a greater drive towards understanding and interpretation in their older age group, and our results corroborate the view that understanding plays an important role in aesthetic appreciation (Leder et al., 2004) but also suggest that this drive for meaning follows a particular time-course which may depend on levels of neurocognitive development, as well as possible influences of experience.

We found significant increases in the recognition of the role of the artist as an agent, namely between ages 4 and 6, and again between ages 6 and 8. In previous research, the ability to consider the role and intentions of the artist has been found to occur in 5- and 7-year-olds and coincides with the development of Theory of Mind (Callaghan and Rochat, 2003). It is notable that reference to the artist increased significantly twice, suggesting that, unlike formal TOM tests which categorize pass (as opposed to fail) on false belief at around age 4 (Baron-Cohen et al., 1985), the spontaneous use of interpretations that testify to a Theory of Mind continue to increase beyond that stage (see also Keysar et al., 2003; Lagattuta et al., 2015). It is also around the age of 7 that children develop an interpretive Theory of Mind (i-TOM) which is the understanding that the same picture can be perceived differently by people (Carpendale and Chandler, 1996) and this may also have been reflected in the children’s increased focus on the intentions of the artist. Such interpretations are in line the view that there is a general widening of perspective-taking ability and reduction in egocentrism (Parsons et al., 1978). As judgment decenters and becomes less egocentric, children can develop a broader, more abstract cognitive style that is associated with expert aesthetic taste (Child, 1965).

It is interesting to note that children at age 10 started using the justification of perceptual fluency, i.e., the experience of the effort of perceiving the artwork (see Reber et al., 1998). This points to a high degree of introspection and metacognitive awareness, as these abilities are necessary for the children to be able to relate their ease of processing the artwork to their level of appreciation. This makes its recognition as a reason for liking artworks by relatively young children quite remarkable. We must add that it was rare for children to articulate this, but it is nevertheless noteworthy that they did at all. It is possible that this is connected to a concept worthy of further research, namely that of conceptual fluency (Alter and Oppenheimer, 2009), which refers to the ease with which meaning can be gleaned from an entity (in our case, an artwork). Paradoxically, it has been observed that adult viewers who have more experience with artwork may value a lack of conceptual fluency (Belke et al., 2015). It would be interesting to study at which time this may onset at slightly later stages of development, as it is conceivable that this may begin during adolescence.

In our study children did not give emotion as a frequent justification (on average 4% overall), and when they did it was significantly more for representational than abstract art, perhaps due to the higher inclusion of facial expressions where emotion is portrayed literally, and thus is more easily understood (Ives, 1984; Jolley et al., 2004). While this significant difference is predicted by association theory, the low level of usage runs counter to the central role that emotions are believed to have in the aesthetic experience (Cupchik and Laszlo, 1992; Leder et al., 2004; Belke et al., 2010; Else et al., 2015). Moreover, Schabmann et al. (2015) found emotion was important in aesthetic evaluations at all ages but more so for younger children, with older children’s evaluation becoming increasingly cognitively based and knowledge-driven. A probable reason for this apparent discrepancy is that, in our study, any reference to emotion was spontaneous, and just because children did not mention emotion in their justifications, does not necessarily mean that emotions did not play a role in their evaluations. As demonstrated by Schepman et al. (2015b), a participant’s thoughts in response to an artwork may not explicitly show any emotional content, but they do have clear and strong emotional relevance to those individuals when they are asked to rate the valence of those thoughts (see also Augustin et al., 2012). In Schabmann’s work the rating of emotion was specifically elicited by a rating scale, while in our study it was only recorded if children mentioned it spontaneously during their justification. We suggest, therefore, that emotions could easily have played a role in our study but children may be less likely to report the role of emotions spontaneously, perhaps because of lower levels of metacognition, difficulties in articulating emotional influences (see e.g., Mayer et al., 1990; Harris, 2008) or because other more salient influences, such as subject matter, came to mind first. For example, children as young as five can be sensitive to emotions expressed in art if they are provided with a set of verbal labels from which to make their decision (e.g., Carothers and Gardner, 1979; Blank et al., 1984) and while children may not spontaneously refer to emotions when matching pictures on the basis of mood until age 11, they can do this at age five when explicitly instructed to do so (Jolley and Thomas, 1995).

To summarize, it is clear from the data that different justifications follow a different developmental time-course, with attributes such as color and subject matter being present at all ages but with understanding and interpretation, perceptual fluency, and reference to the artist gaining in importance with increasing age. This might suggest that certain basic attributes such as color and subject matter are key to aesthetic appreciation and of primary importance at all ages, possibly because they rely less on a particular level of cognitive development. This developmental profile of the children’s justifications, showing a focus on basic perceptual analysis at early ages and an increased role of cognition and understanding at older ages, corresponds to the organization of Leder et al.’s (2004) stage model of aesthetic experience. Perhaps the stages of aesthetic experience described by Leder et al. (2004), have, to an extent, been determined by the stages of neurocognitive development in children. Young children are strongly driven by the visual properties of the artwork, features which remain important at older ages, but with increased age and cognitive development other cognitive processes come into play, which drive the need to understand the artwork. The combination of complete cognitive development, and extensive experience and knowledge of art, enables individuals to process an artwork in different ways, attending to its structural properties, meaning, and style, to reach a richer aesthetic experience (Winston and Cupchik, 1992; Leder et al., 2004; Cupchik et al., 2009).

### Future Neuroscientific Studies

While it has been suggested that understanding the neural mechanisms of aesthetic processing in children may be challenging (see e.g., Nieminen et al., 2011, section 7), our work documenting reasons given for the liking of artwork suggests potential fruitful lines of investigation for future neuro-aesthetic studies. It would seem particularly useful to examine whether, in the viewing of abstract artworks, color-processing areas of the visual cortex are differently engaged than in the viewing of representational artwork (see e.g., Zeki and Marini, 1998). A further fruitful line would be to examine the role of semantic processing in the viewing of artworks, as it would seem that greater activation of visual semantic areas (e.g., left occipito-temporal areas) should be expected during the viewing of representational artworks (Rossion et al., 2000), while activation in these areas may also indicate attempts to attribute meanings to abstract art, as demonstrated by the children in our study and in adults by Schepman et al. (2015a).

## Conclusion

The results support the view that shared liking for artworks starts to emerge around 8 years of age, for representational art only. This is compatible with observations from non-expert adult observers (Vessel and Rubin, 2010; Schepman et al., 2015b), who also show significantly greater convergence in liking for representational than abstract artwork. This is the first time that this has been shown in children. The finding is compatible with an interpretation that shared meanings, associations, and their associated attributions of positive and negative valence emerge at this time. An important implication from our work is that the processes underlying the convergence in aesthetic appreciation may have a wider role in driving shared liking to other items in the world. Our results also show that children are able to provide justifications for their evaluations from a very young age, but are able to do this in more complex and sophisticated ways as they get older. As with non-expert adults, subject matter seems to dominate justifications for representational artwork, while color and formal artistic properties dominate those for abstract art. Overlaying these dominant categories, the art interpretations gain in richness as children get older, showing evidence of newly acquired cognitive and meta-cognitive abilities, such as Theory of Mind, that are likely to be a product of general neurocognitive development, with likely further influences from education and cultural exposure. The results provide a rich overview of the influences on aesthetic preference in children throughout the primary school age.

## Author Contributions

PR, JK and AS developed the idea for the research and developed the materials. JK collected the data. JK entered the rating data, and JL and AL transcribed and coded the verbal data. AS analyzed the data. PR, JK and AS co-wrote the manuscript, and JL and AL commented on the manuscript.

## Conflict of Interest Statement

The authors declare that the research was conducted in the absence of any commercial or financial relationships that could be construed as a potential conflict of interest.
