# Supplementary material for: The Development of Shared Liking of Representational but not Abstract Art in Primary School Children and Their Justifications for Liking
Source: Front Hum Neurosci. 2016 Feb 5;10:21. doi: 10.3389/fnhum.2016.00021 (PMC4743399; doi:10.3389/fnhum.2016.00021)
Supplement: Supplementary file 1 [file Data_Sheet_1.docx]

**Appendix: Artworks used in the study**

**A. Representational artworks**

1: Kevin Heaney: Houses Granite Montana <http://fineartamerica.com/featured/houses-granite-montana-kevin-heaney.html>

2: Ian Sheldon: Peeling Wallpaper <http://www.fine-art.com/art-93431/ian-sheldon/peeling-wallpaper>

3: David Wade: Streamside <http://dart.fine-art.com/art-29992/david-wade/streamside>

4: Bruce Greene: Under the Indian Blanket <http://www.fine-art.com/art-138220/bruce-greene/under-the-indian-blanket>

5: Mark Peterson: '55 T-Bird <http://www.fine-art.com/art-133493/mark-peterson/%2755-t-bird>

6: Jay Kemp: Return to Sender <http://www.fine-art.com/art-99020/jay-kemp/return-to-sender-%28a/p%29>

7: Sergio Zampieri: Autumn Light <http://www.absolutearts.com/cgi-bin/portfolio/art/your-art.cgi?login=sergiozampieri&title=Autumn_light-1289849983t.jpg>

8: Albert Edelfelt: Boys Playing on the Shore <http://en.wikipedia.org/wiki/Albert_Edelfelt#mediaviewer/File:Albert_Edelfelt_-_Boys_Playing_on_the_Shore_-_Google_Art_Project.jpg>

9: Jean Smith: Laugher #4 <http://jeansmithartist.com/wp-content/gallery/laughter-project/laughter4.jpg>

10: Paul Dixon: Ups and Downs <http://affordablebritishart.co.uk/details.php?pid=4611>

**B. Abstract artworks**

11: Boi K' Boi: Mah Abstract Colors Niamh <http://c300221.r21.cf1.rackcdn.com/mah-abstract-colors-niamh-1342576271_b.jpg>

12: Unknown Artist: Ode to Miro <http://www.inkweb.org/gallery.htm>

13: Mystral Casterial: Kandinsky Tribute <http://www.deviantart.com/art/Watercolor-Kandinsky-tribute-175555409>

14: Elizabeth Urabe: In God’s Hands <http://www.arthit.ru/abstract/0079/abstract-art-11.html>

15: Stephanie Kordan Dardashti: Desire Red <http://dart.fine-art.com/art-72046/stephanie-kordan-dardashti/desire-red>

16: Mauren Greenwood: Indulgence <http://www.mpgart.co.uk/abstrIN.htm>

17: Brice Marden: Cold Mountain <http://abstract-art.com/abstraction/l3_more_artists/ma86b_marden.html>

18: ScentOfBlood: Kandinsky Tribute <http://www.deviantart.com/art/Kandinsky-Tribute-117912491>

19: Ingrid Claessen: Nature Green Yellow White <http://www.ingridclaessen.nl/kunstwerk/13179584_natuur+groen+geel+wit.html#.VK5WXtkysow>

20: Ingrid Claessen: No4 <http://www.ingridclaessen.nl/kunstwerk/14892783_no4-100x100cm2011.html#.VK5W4Nkysow>
